# Major Adverse Cardiovascular Events Following Coronary Artery Stenting by History of Hypertensive Disorder of Pregnancy

**DOI:** 10.1161/JAHA.124.035448

**Published:** 2024-10-11

**Authors:** Omar Sigurvin Gunnarsson, Mats Pihlsgård, Moa Handmark, Giovanna Sarno, Isabel Gonçalves, Simon Timpka

**Affiliations:** ^1^ Perinatal and Cardiovascular Epidemiology, Lund University Diabetes Centre, Clinical Sciences Malmö Lund University Malmö Sweden; ^2^ Department of Obstetrics and Gynecology Skåne University Hospital Lund, Malmö Sweden; ^3^ Department of Medical Sciences, Cardiology and Uppsala Clinical Research Center Uppsala University Uppsala Sweden; ^4^ Department of Cardiology and Cardiovascular Research Translational Studies, Clinical Sciences Malmö Lund University Malmö Sweden

**Keywords:** cardiovascular disease, complications, MACE, PCI, preeclampsia, Epidemiology, Secondary Prevention, Coronary Artery Disease

## Abstract

**Background:**

A history of hypertensive disorders of pregnancy is associated with at least twice the risk of incident ischemic heart disease. Whether the long‐term outcome following treatment with coronary artery stenting is associated to the history of hypertensive disorders of pregnancy is unknown.

**Methods and Results:**

We included 8364 women (age ≤65 years) undergoing first coronary artery stenting 2006 to 2022 following their first delivery in 1973 or later, linking nationwide data on percutaneous coronary intervention and delivery history. In total, 1122 women (13.4%) had a history of hypertensive disorders of pregnancy. The main outcome, a major adverse cardiovascular event, was defined as any incident myocardial infarction, ischemic heart disease, cardiac arrest, arrhythmias, angina pectoris, heart failure, cerebral infarction, or sudden death. During a median follow‐up time of 5 years, 258 women with a history of hypertensive disorders of pregnancy had a major adverse cardiovascular event, compared with 1465 women without a history of hypertensive disorders of pregnancy (23% versus 20.2%, *P*=0.028 for log‐rank test). Estimates adjusted for patient‐ and procedural characteristics in proportional hazards regression models suggested that the hazard rate increased only after 4–8 years of follow‐up (hazard ratio [HR], 1.36 [95% CI, 1.05–1.78]) and was primarily driven by women with history of gestational hypertension (HR, 2.15 [95% CI, 1.49–3.11]).

**Conclusions:**

Women with a history of hypertensive disorders of pregnancy have an increased risk of a major adverse cardiovascular event following coronary artery stenting compared with other parous women. A history of hypertensive disorders of pregnancy warrants further attention in the secondary prevention setting of coronary artery disease.

**Nonstandard Abbreviation and Acronym**
MACE: major adverse cardiovascular event

**Clinical Perspective**

**What Is New?**

- It has been unclear if a history of hypertensive disorders of pregnancy (eg, preeclampsia) increases the risk of a major adverse cardiovascular event after coronary artery stenting.
- In this nationwide register‐based cohort study, a history of hypertensive disorders of pregnancy increased the risk of a major cardiovascular event 8 years after coronary artery stenting, primarily in women with history of gestational hypertension.

**What Are the Clinical Implications?**

- A history of hypertensive disorders of pregnancy warrants further attention in the secondary prevention setting of coronary artery disease.

Coronary artery disease is the leading cause of death from cardiovascular disease.^1^ Epidemiological studies during the past 2 decades have shown that a history of preeclampsia and other forms of hypertensive disorders of pregnancy doubles the risk of coronary artery disease 5 to 15 years after delivery.^2^, ^3^ The severity, time of onset, and recurrence of hypertensive disorders of pregnancy seem to play a significant role, each factor individually further increasing the risk of cardiovascular diseases and cardiovascular death.^4^, ^5^, ^6^ Recently, it has also been shown that women with a history of hypertensive disorder of pregnancy have microvascular dysfunction up to 30 years after pregnancy, suggesting that for instance endothelial damage or other factors might function as a link to the increased risk of cardiovascular disease in these women.^7^

Some studies suggest that women have increased risks of major adverse cardiovascular events (MACEs) following percutaneous coronary intervention (PCI) compared with men.^8^, ^9^, ^10^ A feasible strategy to help abrogate these disparities is to identify female‐specific factors associated with higher risk of MACEs following treatment. We and others have recently shown that women with a history of hypertensive disorders of pregnancy have increased progression of coronary atherosclerosis, increased vascular age, and evidence of a different pattern of atherosclerosis in the coronary arteries compared with women without a history of hypertensive disorders of pregnancy.^11^, ^12^ The extent to which women with a history of hypertensive disorder of pregnancy have similar outcomes following coronary artery stenting compared with parous women without a history of hypertensive disorder of pregnancy is less known. Our hypothesis was that a history of hypertensive disorders of pregnancy would be associated with a higher risk of a MACE following coronary artery stenting.

To address the gap in knowledge, the aim of this study was to evaluate the risk of a MACE following coronary artery stenting by a history of hypertensive disorder of pregnancy in young and middle‐aged women. To do so, we used comprehensive registers on deliveries and coronary artery stenting from Sweden.

## METHODS

Because of the sensitive nature of the data collected for this study, requests to access the data from qualified researchers trained in human subject confidentiality protocols may be sent to the Swedish Board of Health and Welfare at socialstyrelsen@socialstyrelsen.se, Statistics Sweden at scb@scb.se, and the SWEDEHEART/SCAAR (Swedish Web‐System for Enhancement and Development of Evidence‐Based Care in Heart Disease. Evaluated According to Recommended Therapies/Swedish Coronary Angiography and Angioplasty Registry) Registry at datauttag@ucr.uu.se.

We conducted a population‐based cohort study of all women 65 years and younger in Sweden, who had their first delivery registered in the Swedish Medical Birth Register 1973 or later and underwent their first coronary artery stenting between January 1, 2006 and May 12, 2022 as identified through the SWEDEHEART or SCAAR registry. The register data were individually linked by using the Swedish personal identification number. Women with previous revascularization (PCI or coronary artery bypass graft) were excluded (n=459). This study was approved by the Regional Ethical Review Board in Lund, Sweden (dnr 2015/792) and the Swedish Ethical Review Authority (2021‐04863). All patients consented to be included in the SWEDEHEART/SCAAR registry.

The Swedish Medical Birth Register includes >98% of all deliveries in Sweden since 1973. The register includes prospectively collected information on demographic data, reproductive history, and information from the pregnancy, delivery, and neonatal period using standardized prenatal, obstetric, and neonatal records.^13^ The primary exposure was a history of hypertensive disorder in any pregnancy, defined by having received any qualifying *International Classification of Diseases* (*ICD*) diagnoses. During the time span of the study (1973–2022), diagnoses were obtained and registered in the Swedish National Medical Birth Register according to *ICD*, in either the *Eighth Revision* (*ICD‐8*), *Ninth Revision* (*ICD‐9*), or *Tenth Revision* (*ICD‐10*). *ICD‐8* was used during the years 1973 to 1986, *ICD‐9* during the years 1987 to 1996, and *ICD‐10* from 1997 forward. Diagnoses were set according to clinical practice at time of diagnosis and preexisting hypertension was also registered early in pregnancy.

In subgroup analyses, women were also further categorized; gestational hypertension (*ICD‐8* codes: 637.01; *ICD‐9* codes: 642B, 642C, 642D, and 642X; *ICD‐10* codes: O13) and preeclampsia developing at gestational week 20+0 or later (*ICD‐8* codes 637.03, 637.04, 637.09, 637.10, and 637.99; *ICD‐9* codes 642E, 642F, 642H, and 642G; *ICD‐10* codes O11, O14, and O15). Furthermore, preeclampsia was divided into term preeclampsia (delivery ≥37 + 0 weeks of gestation) and preterm preeclampsia (delivery <37 + 0 weeks of gestation). Since the 1980s, almost all pregnant women in Sweden have been offered ultrasonographic measurements of gestational age in the late first or early second trimester and that was the gestational age used in our study.^13^ If ultrasound dating was not available, gestational age was typically calculated from last menses, or if this was not available, by clinical estimation. Women with more than 1 type of hypertensive diagnosis were hierarchically classified according to the most severe type of hypertensive disorder (gestational hypertension as the least severe, then term preeclampsia with delivery ≥37+0 weeks of gestation followed by preterm preeclampsia with delivery <37+0 weeks of gestation as the most severe).

Demographic and procedural factors were collected from the SWEDEHEART registry. The SWEDEHEART registry is a national registry established in December 2009 by merging 4 previous registries and contains information on all patients hospitalized for acute coronary syndrome or undergoing coronary or valvular intervention for any indication and has a very high coverage.^14^ Information on women undergoing stenting before December 2009 was gathered from the SCAAR registry. SCAAR holds data on consecutive patients from all 29 centers performing coronary interventions and angiography in Sweden before 2009, when it merged into SWEDEHEART.^15^

The primary outcome was MACE from 30 days to 8 years from first coronary artery stenting. We started follow‐up after 30 days to eliminate the risk for including events that had led to the index procedure. A MACE was defined according to *ICD‐10* diagnostic codes as registered in the Swedish registry on heart intensive care (SWEDEHEART, RIKS‐HIA [Register of Information and Knowledge About Swedish Heart Intensive Care Admissions]) comprising data from all 74 hospitals in Sweden receiving patients with acute heart disease, the Swedish In‐Patient register, and the Swedish Cause of Death register. Diagnoses included in the definition of MACE were myocardial infarction and ischemic heart disease, cardiac arrest and arrhythmias, angina pectoris, heart failure, cerebral infarction, and sudden death (all *ICD‐10* codes used are listed in Table S1). Patients assigned with any of the defined diagnoses as either the main diagnosis or first or second contributing diagnosis >30 days from the index procedure were considered to have an outcome event. We truncated follow‐up at 8 years after coronary artery stenting due to limited number of patients with data beyond that time point and to better study the outcome of treatment rather than overall atherosclerotic progression or effect of aging. We checked the proportional hazards assumption using graphical methods and tests of Schoenfeld residuals. When we checked the model with follow‐up over 8 years for the proportional hazards' assumptions graphically, we noted clear indication of nonproportionality over time. To address this, we conducted a landmark analysis^16^ for our main modeling, with a landmark for first follow‐up at 4 years after the index procedure and a second analysis on the time span of 4 to 8 years after the index procedure. Right‐censoring during follow‐up occurred at 8 years of follow‐up, end of follow‐up in 2022, migration out of Sweden, or death, whichever came first.

### Statistical Analysis

Descriptive data are presented as numbers and percentages or medians and interquartile ranges as appropriate. Comparison of prevalence was made with women without history of hypertensive disorder of pregnancy. To semidescriptively analyze associations between a history of hypertensive disorder of pregnancy and MACE, we constructed cumulative incidence curves.

We identified relevant covariables in the literature and constructed Cox regression models additionally adjusted for covariates, yielding hazard ratios (HR) with 95% CI. Model I included age. Model II was additionally adjusted for patient‐related characterizes at stenting (diabetes, hypertension, hyperlipidemia, body mass index, smoking status, history of stroke or myocardial infarction), and model III was additionally adjusted for procedural covariables (year of procedure, indication for PCI, balloon dilation, number of stents, multivessel disease, total stent length, the smallest diameter of any stent, and if any bare metal stent was inserted). Cox regression modeling was also performed specifically for incident myocardial infarction following stenting, with the same analytical approach as in the main analysis. We performed multiple imputation to impute missing data on a patient level according to the method description of Rubins^17^ by using chained equations with 20 data sets (via PROC MI and PROC MIANALYSE analysis) to account for missing data (Table 1).

**Table 1:** Cohort Characteristics at First Coronary Artery Stenting Summarized by History of Hypertensive Disorders of Pregnancy

		Subgroups of HDP (n=1122)			Non‐HDP (n=7242)
Characteristic	HDP (n=1122)	GH (n=313)	Term PE (n=558)	Preterm PE (n=251)	
Age, y±SD	54.4±7.1	56.2±6.3	54.6±6.8	51.9±8	56±6.3
Body mass index, kg/m^2^ ±SD	29.5±5.8	29.5±5.8	29.7±5.8	28.9±5.8	27.8±5.4
(Missing n, %)	(155,13.8)	(48,15.3)	(69,12.4)	(38,15.1)	
Diabetes, n (%)	290 (25.9)	78 (24.9)	127 (22.8)	85 (33.8)	1080 (14.9)
(Missing n, %)	(7, 0.6)	(4, 1.3)	(3, 0.5)		(31–0.4)
Smoking status, n (%)					
(Missing n, %)	(47, 4.2)	(16, 5.1)	(18, 3.2)	(135.2)	(262, 3.6)
Never	495 (44.1)	141 (45)	238 (42.7)	116 (46.2)	2300 (31.8)
Quit	295 (26.3)	75 (24)	162 (29)	58 (23)	1724 (23.8)
Current	332 (29.6)	97 (31)	158 (28.3)	77 (30.7)	3218 (44.5)
Hypertension, n (%)	696 (62)	193 (61.7)	343 (61.5)	160 (63.8)	3071 (42.4)
(Missing n, %)	(10, 0.8)	(5, 1.6)	(5, 0.9)		(94, 1.3)
Dyslipidemia, n (%)	383 (34.1)	102 (32.6)	185 (33.2)	96 (38.3)	2105 (29.1)
(Missing n, %)	(19, 1.6)	(9, 2.8)	(8, 1.4)	(2, 0.8)	(107, 1.5)
Parity, n (%)					
1	194 (17.3)	48 (15.3)	92 (16.5)	54 (21.5)	1558 (21.5)
2–3	777 (69.3)	220 (70.3)	389 (69.7)	168 (66.9)	4950 (68.4)
4 or more	151 (13.5)	45 (14.4)	77 (13.8)	29 (11.6)	734 (10.1)
Prior MI, n (%)	46 (4.1)	15 (4.8)	14 (2.5)	17 (6.8)	255 (3.5)
(Missing n, %)	(15, 1.3)	(8, 2.6)	(5, 0.9)	(2, 0.8)	(80, 1.1)
Prior stroke, n (%)	31 (2.8)	6 (1.9)	14 (2.5)	11 (4.4)	143 (2)
Multivessel disease, n (%)	400 (35.7)	116 (37.1)	189 (33.9)	95 (38)	2250 (31.1)
Any bare metal stent used, n (%)	236 (21)	80 (25.6)	110 (19.7)	46 (18.3)	1573 (21.7)
Percutaneous coronary intervention indication, n (%)					
STEMI	373 (33.2)	117 (37.4)	169 (30.3)	87 (34.7)	2697 (37.2)
Non–STEMI	223 (19.9)	57 (18.2)	118 (21.2)	48 (19.1)	1379 (19.1)
Unstable CAD	305 (27.2)	78 (24.9)	165 (29.6)	62 (24.7)	1916 (26.5)
Stable CAD	173 (15.4)	52 (16.6)	82 (14.7)	39 (15.5)	995 (13.8)
Other	48 (4.3)	9 (2.9)	24 (4.3)	15 (6)	255 (3.5)

Missing data presented as n (%) with relevant characteristic. CAD indicates coronary artery disease; GH, gestational hypertension; HDP, hypertensive disorder of pregnancy; MI, myocardial infarction; PE, preeclampsia; and STEMI, ST‐segment–elevation MI.

Statistical significance was set at the 2‐tailed *P* value <0.05. Statistical analyses were performed using SAS 9.4 (SAS Institute, Cary, NC, USA.).

## RESULTS

**Figure 1:**
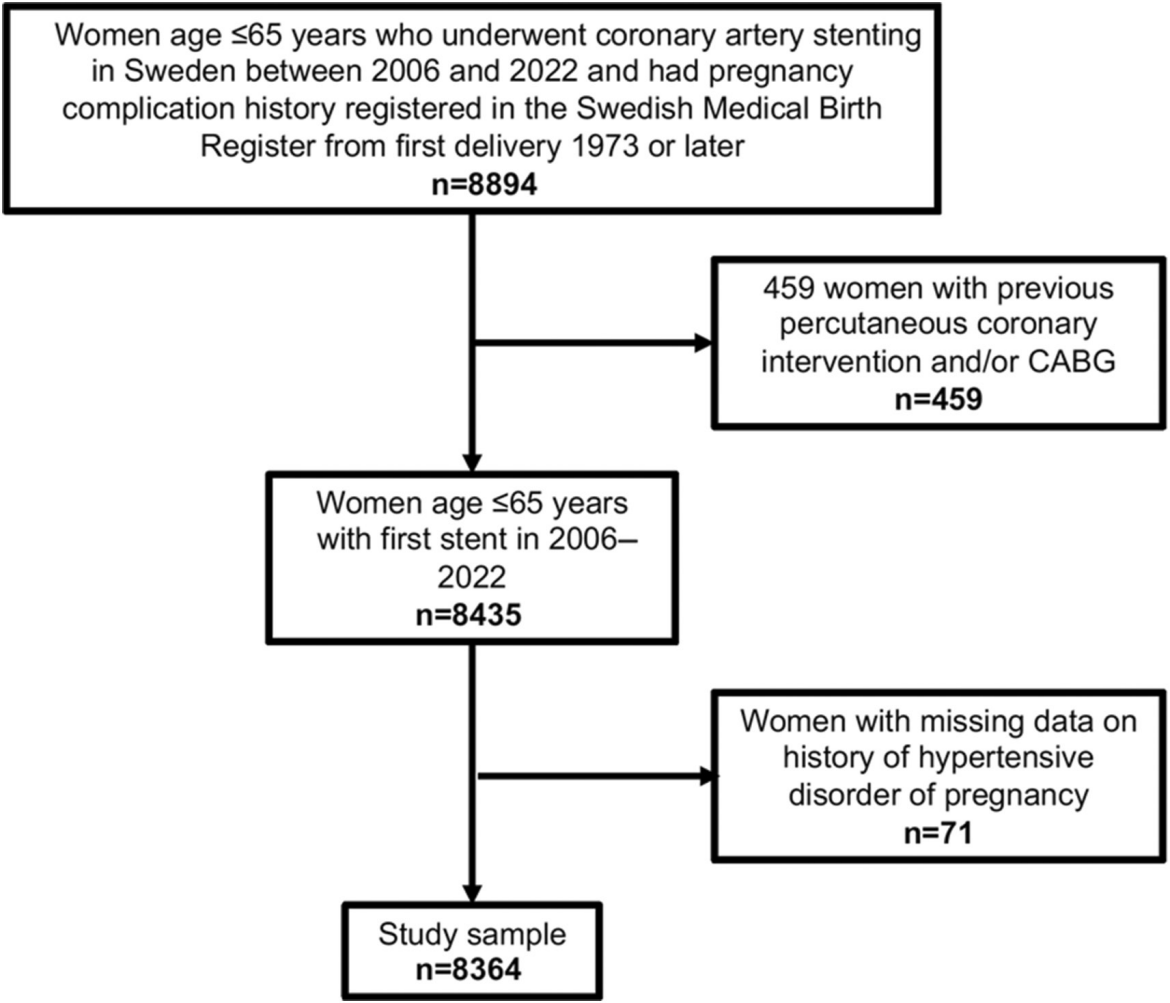
Flow chart of study sample. CABG indicates coronary artery bypass grafting.

In total, the study sample consisted of 8364 women age ≤65 years, 1122 of whom (13.4%) had a history of hypertensive disorder of pregnancy (Figure 1). At the time of coronary artery stenting, women with a history of hypertensive disorder of pregnancy had nominally higher body mass index and were more likely to have a history of diabetes, hypertension, and dyslipidemia before index procedure, as well as multivessel disease (Table 1). The women without a history of hypertensive disorder of pregnancy were more likely to be smokers at index procedure. Women with a history of hypertensive disorder of pregnancy were also younger at the time of the index procedure as seen in Table 1. Women with a history of preterm preeclampsia were approximately 4 years younger when undergoing the procedure than women without a history of hypertensive disorder of pregnancy.

**Figure 2:**
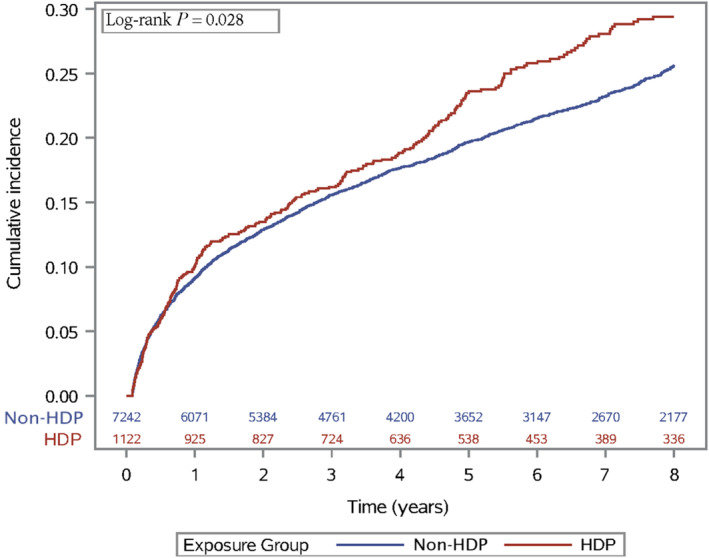
Cumulative incidence plot of major adverse cardiovascular events following first coronary artery stenting in women by history of hypertensive disorders of pregnancy. Major adverse cardiovascular event was defined as myocardial infarction, ischemic heart disease, cardiac arrest, arrhythmias, angina pectoris, heart failure, cerebral infarction, or sudden death. HDP indicates hypertensive disorder of pregnancy; and non‐HDP; no history of hypertensive disorder of pregnancy.

Median follow‐up time was 5 years (interquartile range 1.9–8.0 years). During 39 637 person‐years of total follow‐up, 258 women (23.0%) with a history of hypertensive disorder of pregnancy had a MACE, compared with 1465 (20.2%) of women without a history of hypertensive disorder of pregnancy in the first 8 years after stenting. This corresponds to 49.7 events per 1000 person‐years and 42.5 events per 1000 person‐years, respectively. As shown in Figure 2, the unadjusted incidence for MACE after coronary artery stenting was higher in women with history of hypertensive disorder of pregnancy (log‐rank *P*=0.028).

As shown in Table 2, estimates from proportional hazards regression modeling suggested a small risk increase in age‐adjusted (HR, 1.14 [95% CI, 1.00–1.30]) but not in fully adjusted models (HR, 1.03 [95% CI, 0.90–1.18]) for women with a history of a hypertensive disorder of pregnancy. Subgroup analysis showed increased risk for women with history of preterm preeclampsia in age‐adjusted models (HR, 1.35 [95% CI, 1.04–1.75]) but this difference was abrogated in fully adjusted models.

**Table 2:** Major Adverse Cardiovascular Events Following Coronary Artery Stenting by History of Hypertensive Disorders of Pregnancy

			Subgroups of HDP (n=1122)						Non‐HDP (n=7242)
Model	HDP (n=1122)		GH (n=313)		Term PE (n=558)		Preterm PE (n=251)		
	HR (95% CI)	*P* value	HR (95% CI)	*P* value	HR (95% CI)	*P* value	HR (95% CI)	*P* value	
Model I	1.14 (1.00–1.30)	0.05	1.09 (0.87–1.37)	0.46	1.08 (0.90–1.30)	0.39	1.35 (1.04–1.75)*	0.02*	1.00 (reference)
Model II	1.05 (0.92–1.20)	0.50	1.02 (0.82–1.28)	0.84	1.01 (0.84–1.22)	0.88	1.16 (0.89–1.50)	0.28	1.00 (reference)
Model III	1.03 (0.90–1.18)	0.69	1.01 (0.81–1.27)	0.92	1.00 (0.83–1.21)	0.96	1.10 (0.85–1.44)	0.47	1.00 (reference)

Model I included a history of hypertensive disorder of pregnancy and age at the index coronary artery stenting (continuous). Model II additionally contains baseline variables diabetes (yes/no); hypertension (yes/no); dyslipidemia (yes/no); body mass index (continuous); smoking status (never smoker, exsmoker, current smoker); previous stroke (yes/no); previous MI (yes/no). Model III additionally includes year of procedure (2006–2009, 2010–2013, 2014–2017, 2018–2022); indication for coronary artery stenting (STEMI, non–STEMI, unstable coronary artery disease, stable coronary artery disease, other); procedure type (PCI, PCI ad hoc); number of stents (1, 2, ≥3); multivessel disease (yes/no); total stent length (continuous); smallest diameter of any stent (continuous); Bare metal stent inserted at procedure (yes/no; if no, then only drug eluting stent was inserted at procedure). Results from a proportional hazards regression, multiple imputation analysis. Major adverse cardiovascular event was defined as myocardial infarction, ischemic heart disease, cardiac arrest, arrhythmias, angina pectoris, heart failure, cerebral infarction, or sudden death. GH indicates gestational hypertension; HDP, hypertensive disorder of pregnancy; HR, hazard ratio; MI, myocardial infarction; PE, preeclampsia; PCI, percutaneous coronary intervention; and STEMI, ST‐segment–elevation MI.

* *P*<0.05.

**Figure 3:**
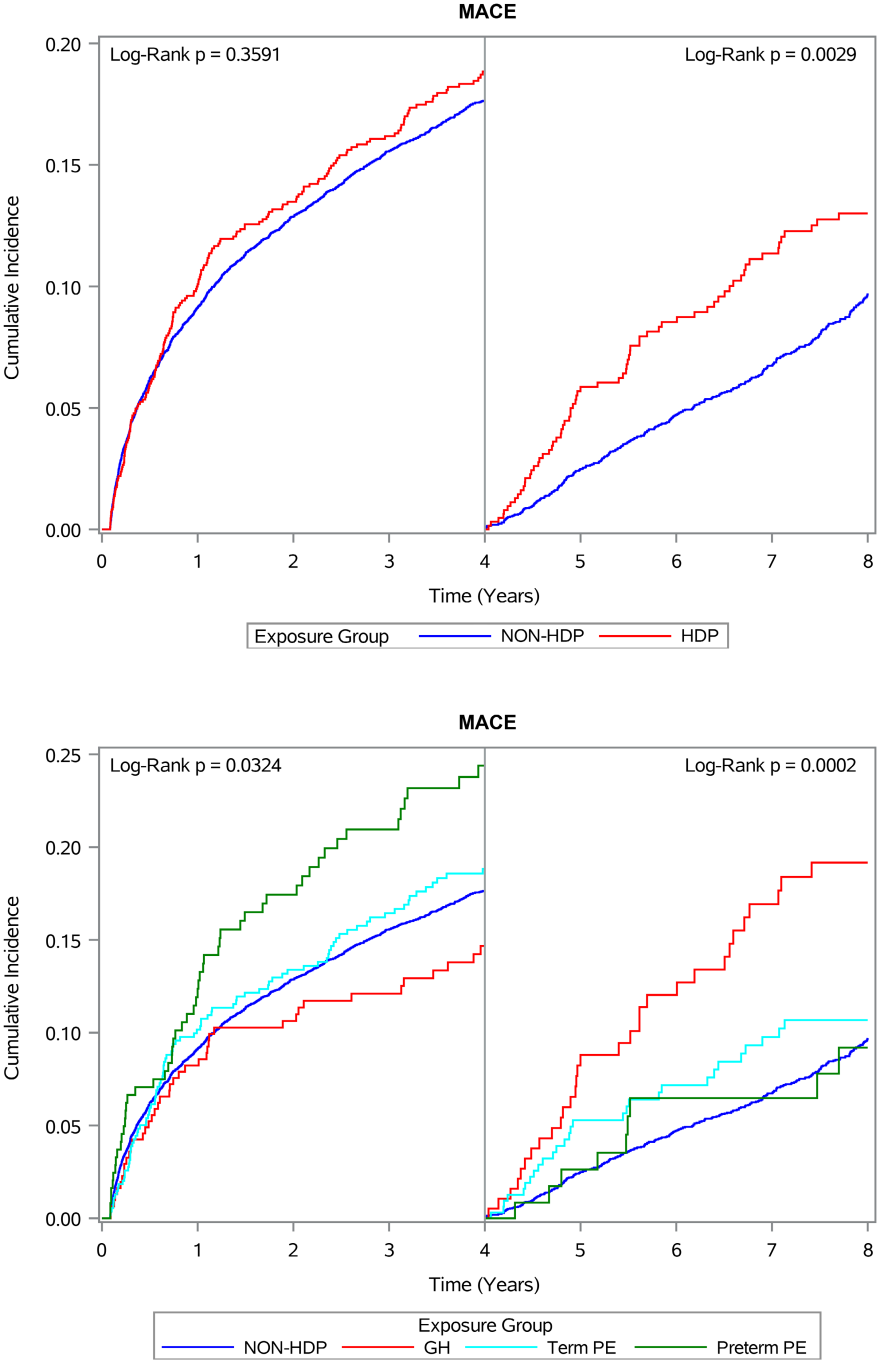
Cumulative incidence plot of major adverse cardiovascular events following first coronary artery stenting in women by history of hypertensive disorders of pregnancy, with landmark analysis at 4 years and 8 years. **Upper** panel: Comparison according to history of hypertensive disorder of pregnancy. **Lower** panel: Comparison according to subgroups of hypertensive disorder of pregnancy. Major adverse cardiovascular event was defined as myocardial infarction, ischemic heart disease, cardiac arrest, arrhythmias, angina pectoris, heart failure, cerebral infarction, or sudden death. GH indicates gestational hypertension; HDP, hypertensive disorder of pregnancy; non‐HDP, no history of hypertensive disorders of pregnancy; and PE, preeclampsia.

Figure 3 shows the results from the landmark analysis, in which the risks of a MACE by hypertensive disorders of pregnancy history during the 2 subsequent 4‐year periods following the first coronary stenting are presented separately. As also shown in Table 3, an increased risk of MACE in women with a history of hypertensive disorders of pregnancy is evident only during the second 4‐year period (HR, 1.34 [95% CI, 1.03–1.74] in the fully adjusted model). Furthermore, subgroup analysis showed that the increased risk was isolated to women with a history of gestational hypertension (HR, 2.12 [95% CI, 1.47–3.07] in the fully adjusted model).

**Table 3:** Major Adverse Cardiovascular Events Following First Coronary Artery Stenting by History of Hypertensive Disorders of Pregnancy Divided Into 2 Periods of Follow‐Up

				Subgroups of HDP (n=1122)						Non‐HDP (n=7242)
Model	Period	HDP (n=1122)		GH (n=313)		Term PE (n=558)		Preterm PE (n=251)		
		HR (95% CI)	*P* value	HR (95% CI)	*P* value	HR (95% CI)	*P* value	HR (95% CI)	*P* value	
Model I	0–4 years	1.05 (0.90–1.23)	0.51	0.79 (0.58–1.07)	0.13	1.05 (0.85–1.30)	0.64	1.44 (1.09–1.90)*	0.01*	1.00 (reference)
	4–8 years	1.46 (1.12–1.90)*	0.005*	2.23 (1.53–3.24)*	<0.001*	1.20 (0.82–1.77)	0.35	0.97 (0.50–1.89)	0.93	1.00 (reference)
Model II	0–4 years	0.97 (0.83–1.13)	0.65	0.74 (0.54–1.00)	0.05	0.99 (0.80–1.22)	0.89	1.22 (0.93–1‐60)	0.15	1.00 (reference)
	4–8 years	1.36 (1.05–1.78)*	0.02*	2.15 (1.49–3.11)*	<0.001*	1.12 (0.76–1.66)	0.56	0.87 (0.44–1.70)	0.68	1.00 (reference)
Model III	0–4 years	0.95 (0.81–1.11)	0.50	0.73 (0.53–0.99)	0.05	0.97 (0.79–1.20)	0.81	1.17 (0.89–1.54)	0.27	1.00 (reference)
	4–8 years	1.34 (1.03–1.74)*	0.03*	2.12 (1.47–3.07)*	<0.001*	1.12 (0.76–1.65)	0.58	0.82 (0.42–1.60)	0.55	1.00 (reference)

Model I included a history of hypertensive disorder of pregnancy and age at the index coronary artery stenting (continuous). Model II additionally contains baseline variables diabetes (yes/no); hypertension (yes/no); dyslipidemia (yes/no); body mass index (continuous); smoking status (never smoker, ex‐moker, current smoker); previous stroke (yes/no); previous MI (yes/no). Model III additionally includes year of procedure (2006–2009, 2010–2013, 2014–2017, 2018–2022); indication for coronary artery stenting (STEMI, non–STEMI, unstable coronary artery disease, stable coronary artery disease, other); procedure type (PCI, PCI ad hoc); number of stents (1, 2, ≥3); multivessel disease (yes/no); total stent length (continuous); smallest diameter of any stent (continuous); bare metal stent inserted at procedure (yes/no; if no, then only drug eluting stent was inserted at procedure). All models are then stratified into periods of follow‐up (0–4 years/4–8 years). Results from a proportional hazards regression, multiple imputation analysis. Major adverse cardiovascular event was defined as myocardial infarction, ischemic heart disease, cardiac arrest, arrhythmias, angina pectoris, heart failure, cerebral infarction, or sudden death. GH indicates gestational hypertension; HDP, hypertensive disorder of pregnancy; HR, hazard ratio; MI, myocardial infarction; PE, preeclampsia; PCI, percutaneous coronary intervention; and STEMI, ST‐segment–elevation MI.

* *P*<0.05.

A more specific subgrouping of the diagnoses leading to a first MACE in participants is presented in Table S2. The proportions were similar in women with and without a history of hypertensive disorders of pregnancy. Analyzing myocardial infarction as an outcome specifically resulted in similar relative incidence as compared with MACE, as shown in Figure S1 and Figure S2. As shown in Table S3, there was an increased risk of myocardial infarction in all models for women with a history of hypertensive disorder of pregnancy. In fully adjusted landmark analysis, women with history of preterm preeclampsia had increased risk of myocardial infarction in the first 4 years following stenting (HR, 1.57 [95% CI, 1.05–2.37]) and women with a history of gestational hypertension after 4 to 8 years (HR, 2.06 [95% CI, 1.19–3.57]), as shown in Table S4.

## DISCUSSION

In this comprehensive nationwide study, the main finding was that women with a history of hypertensive disorder of pregnancy were more likely to have a MACE in the first 8 years after coronary artery stenting. This difference was primarily driven by increased risk of a MACE 4 to 8 years after the index procedure in women with a history of gestational hypertension.

As history of hypertensive disorder of pregnancy has been considered as a failed cardiometabolic “stress test” in the primary prevention setting,^18^, ^19^, ^20^ we hypothesized that we would find a similar association in the secondary prevention setting. In other words, the risk of a second event following stenting would be increased in women with history of hypertensive disorder of pregnancy, and even more so in those women with the more severe form, such as those with a history of preterm preeclampsia. We have previously reported that women with a history of preterm delivery have increased risk for a MACE in the first 4 years after stenting,^21^ which led to the current hypothesis that preeclampsia would further increase the risk.

Many of the factors that mediate impaired cardiometabolic adaptation during pregnancy and cardiovascular disease pathogenesis before and after pregnancy are shared.^22^, ^23^, ^24^ A susceptibility to endothelial dysfunction is seen after a pregnancy complicated by hypertensive disorder of pregnancy^25^ and is also known to be a common factor in the development of coronary artery disease.^26^ Furthermore endothelial dysfunction has been linked to an increased risk of restenosis after PCI.^27^ Based on results from animal models, it has been suggested that women with a history of preeclampsia would also have increased risk of restenosis following PCI^28^ but we did not find any evidence of an increased risk in a recently published human clinical study.^29^

Women with hypertensive disorders of pregnancy have increased progression of coronary atherosclerosis, and this has been confirmed with noninvasive and invasive diagnostics.^12^, ^30^ It has also been shown that the risk of ischemic heart disease increases according to severity of hypertensive disorder of pregnancy, with preterm preeclampsia most closely linked to ischemic heart disease.^4^ In a prospective study on 251 women followed up after acute coronary syndrome, those with a history of preeclampsia were much more likely to have a recurrent episode of acute coronary syndrome than those without a history of preeclampsia in the 12 months following the initial incidence.^31^ A study on 1985 women, with less complete data on patient and procedural characteristics than our study, reported that women with a history of preeclampsia had twice the risk of death and women with recurrent preeclampsia quadruple the risk of death in the first 5 years following coronary artery revascularization compared with women with pregnancies unaffected by hypertensive disorders but no increased risk of MACEs.^32^

In our study the risk for a MACE was increased only during the second 4‐year period of follow‐up in women with a history of hypertensive disorder of pregnancy. In our initial analysis preterm preeclampsia seemed to increase the hazard rate but this did not hold in the adjusted models and only gestational hypertension remained statistically significant.

We have recently shown that women with a history of preeclampsia and gestational hypertension have increased vascular age, which has also been proposed based on the results from a recent hemodynamic study.^11^, ^33^ In a recent study on 391 women aged 65 years and younger with myocardial infarction, women with a history of preeclampsia experienced myocardial infarction 4 years earlier than women without a history of preeclampsia.^34^ In our study, women with a history of hypertensive disorder of pregnancy had more risk factors for cardiovascular diseases (diabetes, hypertension, dyslipidemia, overweight) than women with history of normotensive pregnancies. In subgroup analysis there was a difference in timing of myocardial infarction, where women with history of preterm preeclampsia had increased risk of a myocardial infarction in the first 4 years following stenting and women with history of gestational hypertension 4 to 8 years after stenting. Our findings support that there are partly distinct pathogenetic processes behind different subtypes of hypertensive disorder of pregnancy, as these subsequently are associated with different patterns of cardiovascular disease in affected women following coronary artery stenting.

A possible explanation for our findings is that the increased risk we found in women with a history of gestational hypertension after a follow‐up period of 4 to 8 years is a result of more advanced vascular age, causing an accelerated incidence of a second event even though treatment of the first event was generally successful. It is well known that women with a history of gestational hypertension have an increased risk of chronic hypertension and long‐term maternal risk.^35^, ^36^ With this in mind, another possible explanation could be that women with a history of gestational hypertension have a more severe hypertension, worse treatment effect, more therapy resistance, or less compliance to their antihypertensive treatment following stenting, which would contribute to a higher event rate in the long term. In a recent study in which we quantified coronary artery atherosclerosis using coronary computed tomography angiography, point estimates suggested that women with a history of gestational hypertension had higher prevalence of coronary atherosclerosis. They more frequently also had higher coronary artery calcium score, which might contribute to our understanding of why they have a higher risk of a late MACE following coronary artery stenting.

The main strength of this study is the use of comprehensive and nationwide leveraging data from well‐known and reliable clinical databases.^13^, ^14^, ^37^ With many relevant predictors of MACEs well documented in the study sample, comprehensive adjustment in regression models was possible. Additionally, the follow‐up was quite long compared with other studies, allowing the identification of long‐term complications. However, this study is not without limitations. First, performing many statistical comparisons leads to the risk of type I error, for example, the increased HR reported for women with history of gestational hypertension during the second period should be regarded as an exploratory result. Second, even though the Swedish Medical Birth Registry includes >98% of all deliveries in Sweden, misclassifications may have occurred. Although we adjusted our models for patient and periprocedural factors, we do not have comprehensive data on medications during follow‐up. Still, we do not think it is plausible that prescription drugs would differ substantially by history of hypertensive disorder of pregnancy given that we have accounted for indication and type of procedure and that history of hypertensive disorder of pregnancy was likely not known to the clinician at the time of care. Lastly, in our study we looked at women aged 65 years and younger in a Swedish population without information on ethnicity, which is known to affect the risk for cardiovascular disease^38^ and could limit the generalizability of our results to other populations.

To better understand the clinical utility of tailoring treatment to different subgroups of women by history of hypertensive disorders of pregnancy in coronary disease secondary prevention, larger studies that include data on both hypertensive disorders of pregnancy history and treatment of coronary artery disease are needed.

## CONCLUSIONS

Women with a history of hypertensive disorders of pregnancy have increased risk of MACEs following coronary artery stenting compared with other parous women without that history. A history of hypertensive disorders of pregnancy warrants further attention in the secondary prevention setting of coronary artery disease.

## Sources of Funding

Funding of the work was through a grant from the Swedish Research Council (2019‐02082, 2022‐01771), the Swedish Heart Lung Foundation (20220185), and Lund University (ALF project 2022‐Projekt0112). Isabel Gonçalves has received funding from the Swedish Research Council, the Swedish Heart and Lung Foundation, Skåne University Hospital funds, and Lund University Diabetes Center (Swedish Research Council – Strategic Research Area Exodiab Dnr 2009‐1039, Linnaeus grant Dnr 349‐2006‐23 and the Swedish Foundation for Strategic Research Dnr IRC15‐006). The funders had no role or influence in any of the following: design and conduct of the study; collection, management, analysis, and interpretation of the data; preparation, review, or approval of the article; and decision to submit the article for publication.

## Disclosures

None.

## Supporting information

Data S1
