# Supplementary material for: Major Adverse Cardiovascular Events Following Coronary Artery Stenting by History of Hypertensive Disorder of Pregnancy
Source: J Am Heart Assoc. 2024 Oct 11;13(20):e035448. doi: 10.1161/JAHA.124.035448 (PMC11935595; doi:10.1161/JAHA.124.035448)
Supplement: Supplementary file 1 — Data S1 [file JAH3-13-e035448-s001.pdf]

Supplemental Material

Table S1. ICD codes comprising outcome variable, MACE

Cardiovascular event *(Swedish registry on heart intensive care (SWEDEHEART, RIKS-HIA) and Swedish In-patient register)*

	**ICD 10**	**ICD 9**	**ICD 8**
Acute myocardial infarction	I21	410	410
Subsequent myocardial infarction	I22	411	411
Unstable or unspecified angina	I20.0<br>I20.9	413	413
Cerebral infarction	I63	434	434
Stroke, not specified as hemorrhage or infarction	I64	436	436

Cardiovascular deaths *(Swedish Cause of death register)*

	**ICD 10**
Unstable or unspecified angina	I20.0<br>I20.9
Acute myocardial infarction	I21
Subsequent myocardial infarction	I22
Chronic ischaemic heart disease	I25.0<br>I25.1<br>I25.2<br>I25.5<br>I25.6<br>I25.8<br>I25.9
Cardiac arrest	I46
Sudden death, cause unknown	R96<br>R98<br>R99
Atrial fibrillation and flutter	I48
Ventricular fibrillation and flutter	I49.0
Heart failure	I50
Cerebral infarction	I63

ICD, international classification of diseases

ICD 8 from 1969 to 1986, ICD 9 from 1987 to 1996, ICD 10 from 1996 to now

ICD10 codes are used to define study outcomes.
ICD8 and ICD9 codes are used to define preexisting disease.

Table S2. Specific diagnostic codes set at the time of first incident major adverse cardiovascular event following the index procedure

Diagnosis	History of HDP (n=258 patients with 270 diagnoses)	No history of HDP (n=1465 patients with 1510 diagnoses)
Angina pectoris, n (%)	151 (58.5)	884 (60.3)
Myocardial infarction and ischemic heart disease, n (%)	85 (32.9)	448 (30.6)
Cerebral infarction, n (%)	17 (6.6)	87 (5.9)
Cardiac arrest and arrhythmias, n (%)	8 (3.1)	48 (3.3)
Heart failure, n (%)	7 (2.7)	40 (2.7)
Sudden death, n (%)	2 (0.8)	3 (0.2)

HDP, hypertensive disorder of pregnancy.

Each patient is allowed to contribute with more than one diagnostic code to this table. Diagnosis was obtained from three different databases (Swedish registry on heart intensive care (SWEDEHEART, RIKS-HIA), Swedish In-patient register and Swedish Cause of death register). In total, 4.6% of patients with a history of HDP and 3.1% of patients without a history of HDP had >1 diagnosis of a major adverse cardiovascular event concomitantly registered.

Table S3. Myocardial infarctions following coronary artery stenting by history of hypertensive disorders of pregnancy

			**Subgroups of HDP (n = 1,122)**						
Model	**HDP (n = 1,122)**		**GH (n = 313)**		**Term PE (n = 558)**		**Preterm PE (n = 251)**		**Non-HDP (n = 7,242)**
	HR (95% CI)	p value	HR (95% CI)	p value	HR (95% CI)	p value	HR (95% CI)	p value	
Model I	**1.35 (1.10-1.66)**	**0.04**	1.39 (0.98-1.97)	0.06	1.19 (0.89-1.59)	0.24	**1.68 (1.15-2.46)**	**0.01**	1.00 (reference)
Model II	**1.29 (1.05-1.59)**	**0.02**	1.34 (0.95-1.90)	0.09	1.17 (0.87-1.57)	0.31	**1.51 (1.03-2.22)**	**0.03**	1.00 (reference)
Model III	**1.25 (1.01-1.54)**	**0.04**	1.29 (0.91-1.84)	0.15	1.14 (0.85-1.53)	0.40	1.42 (0.96-2.09)	0.08	1.00 (reference)

BMI, body mass index; BMS, bare metal stent; CI, confidence interval; GH, gestational hypertension; HDP, hypertensive disorder of pregnancy; HR, hazard ratio; NSTEMI, non-ST-elevation myocardial infarction; PE, preeclampsia; PCI, percutaneous coronary intervention; STEMI, ST-elevation myocardial infarction

Model I included a history of hypertensive disorder of pregnancy and age at the index coronary artery stenting (continuous). Model II additionally contains baseline variables diabetes (yes/no); hypertension (yes/no); dyslipidemia (yes/no); BMI (continuous); smoking status (never smoker, ex-smoker, current smoker); previous stroke (yes/no); previous MI (yes/no). Model III additionally includes year of procedure (2006-2009, 2010-2013, 2014-2017, 2018-2022); indication for coronary artery stenting (STEMI, NSTEMI, unstable coronary artery disease, stable coronary artery disease, other); procedure type (PCI, PCI ad hoc); number of stents (1, 2, ≥3); multivessel disease (yes/no); total stent length (continuous); smallest diameter of any stent (continuous); BMS inserted at procedure (yes/no; if no, then only drug eluting stent (DES) was inserted at procedure).

$p<0.05$ presented in bold

Table S4. Myocardial infarctions following coronary artery stenting by history of hypertensive disorders of pregnancy, divided into two periods of follow-up

				**Subgroups of HDP (n = 1,122)**						
Model	Period	**HDP (n = 1,122)**		**GH (n = 313)**		**Term PE (n = 558)**		**Preterm PE (n = 251)**		**Non-HDP (n = 7,242)**
		HR (95% CI)	p value	HR (95% CI)	p value	HR (95% CI)	p value	HR (95% CI)	p value	
Model I	0-4 years	**1.30 (1.02-1.66)**	**0.03**	1.08 (0.67-1.73)	0.75	1.17 (0.82-1.66)	0.38	**1.90 (1.26-2.88)**	**0.002**	1.00 (reference)
	4-8 years	**1.47 (1.02-2.12)**	**0.04**	**2.13 (1.24-3.67)**	**0.01**	1.24 (0.73-2.11)	0.44	1.08 (0.44-2.63)	0.86	1.00 (reference)
Model II	0-4 years	1.23 (0.96-1.58)	0.10	1.02 (0.64-1.63)	0.93	1.14 (0.80-1.63)	0.45	**1.67 (1.11-2.53)**	**0.01**	1.00 (reference)
	4-8 years	**1.45 (1.0-2.11)**	**0.05**	**2.15 (1.25-3.69)**	**0.01**	1.22 (0.71-2.09)	0.46	1.02 (0.41-2.52)	0.96	1.00 (reference)
Model III	0-4 years	1.18 (0.93-1.52)	0.18	0.99 (0.62-1.57)	0.95	1.11 (0.78-1.58)	0.56	**1.57 (1.05-2.37)**	**0.03**	1.00 (reference)
	4-8 years	1.40 (0.96-2.03)	0.08	**2.06 (1.19-3.57)**	**0.01**	1.20 (0.70-2.05)	0.51	0.95 (0.38-2.37)	0.92	1.00 (reference)

BMI, body mass index; BMS, bare metal stent; CI, confidence interval; GH, gestational hypertension; HDP, hypertensive disorder of pregnancy; HR, hazard ratio; NSTEMI, non-ST-elevation myocardial infarction; PE, preeclampsia; PCI, percutaneous coronary intervention; STEMI, ST-elevation myocardial infarction

Model I included a history of hypertensive disorder of pregnancy and age at the index coronary artery stenting (continuous). Model II additionally contains baseline variables diabetes (yes/no); hypertension (yes/no); dyslipidemia (yes/no); BMI (continuous); smoking status (never smoker, ex-smoker, current smoker); previous stroke (yes/no); previous MI (yes/no). Model III additionally includes year of procedure (2006-2009, 2010-2013, 2014-2017, 2018-2022); indication for coronary artery stenting (STEMI, NSTEMI, unstable coronary artery disease, stable coronary artery disease, other); procedure type (PCI, PCI ad hoc); number of stents (1, 2, ≥3); multivessel disease (yes/no); total stent length (continuous); smallest diameter of any stent (continuous); BMS inserted at procedure (yes/no; if no, then only drug eluting stent (DES) was inserted at procedure).
All models are then stratified into periods of follow-up (0-4 years/4-8 years).

$p<0.05$ presented in bold

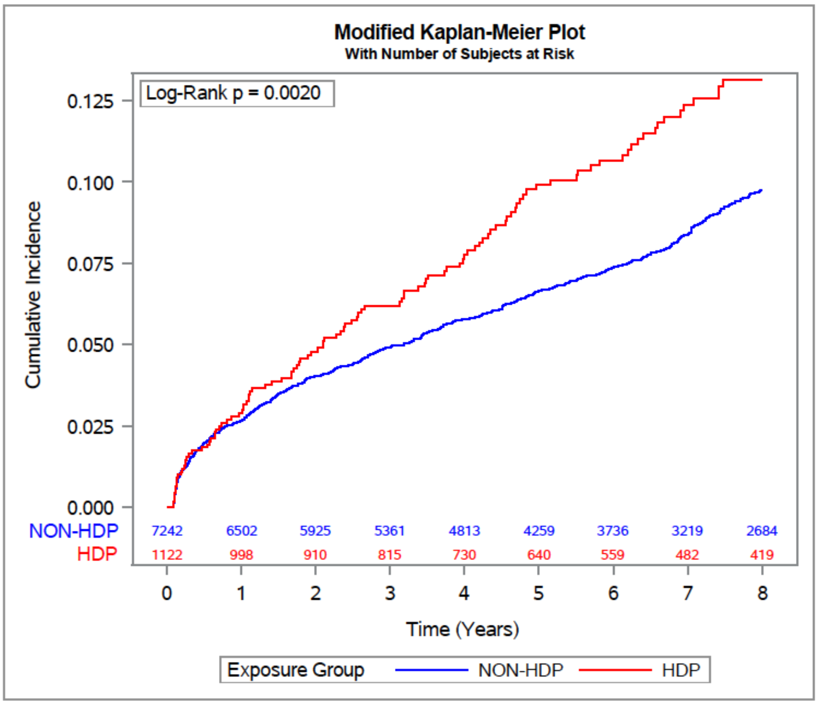

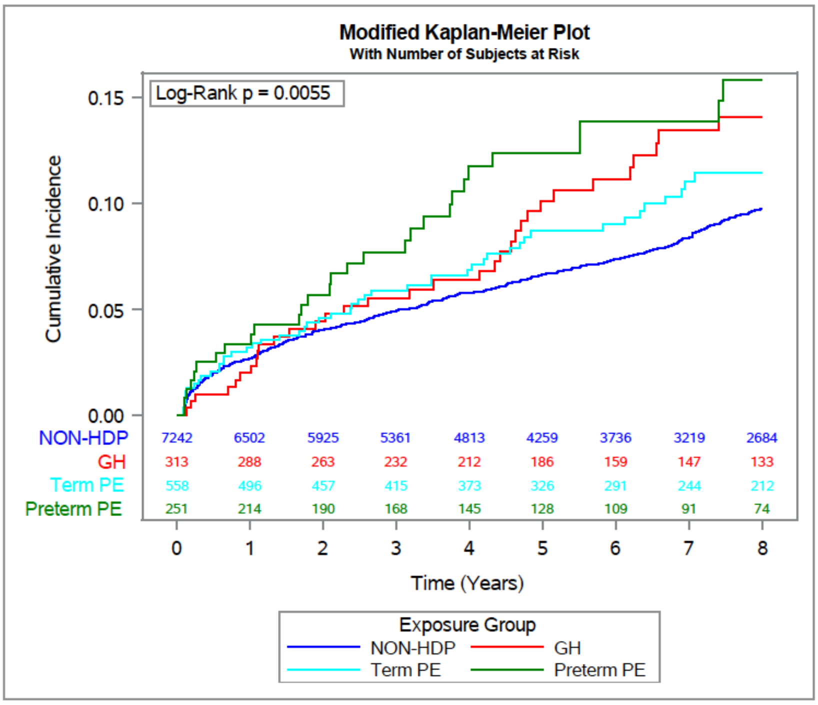

Figure S1. Cumulative incidence plot of myocardial infarctions following first coronary artery stenting in women by history of hypertensive disorders of pregnancy.

HDP, hypertensive disorder of pregnancy; non-HDP; no history of hypertensive disorder of pregnancy

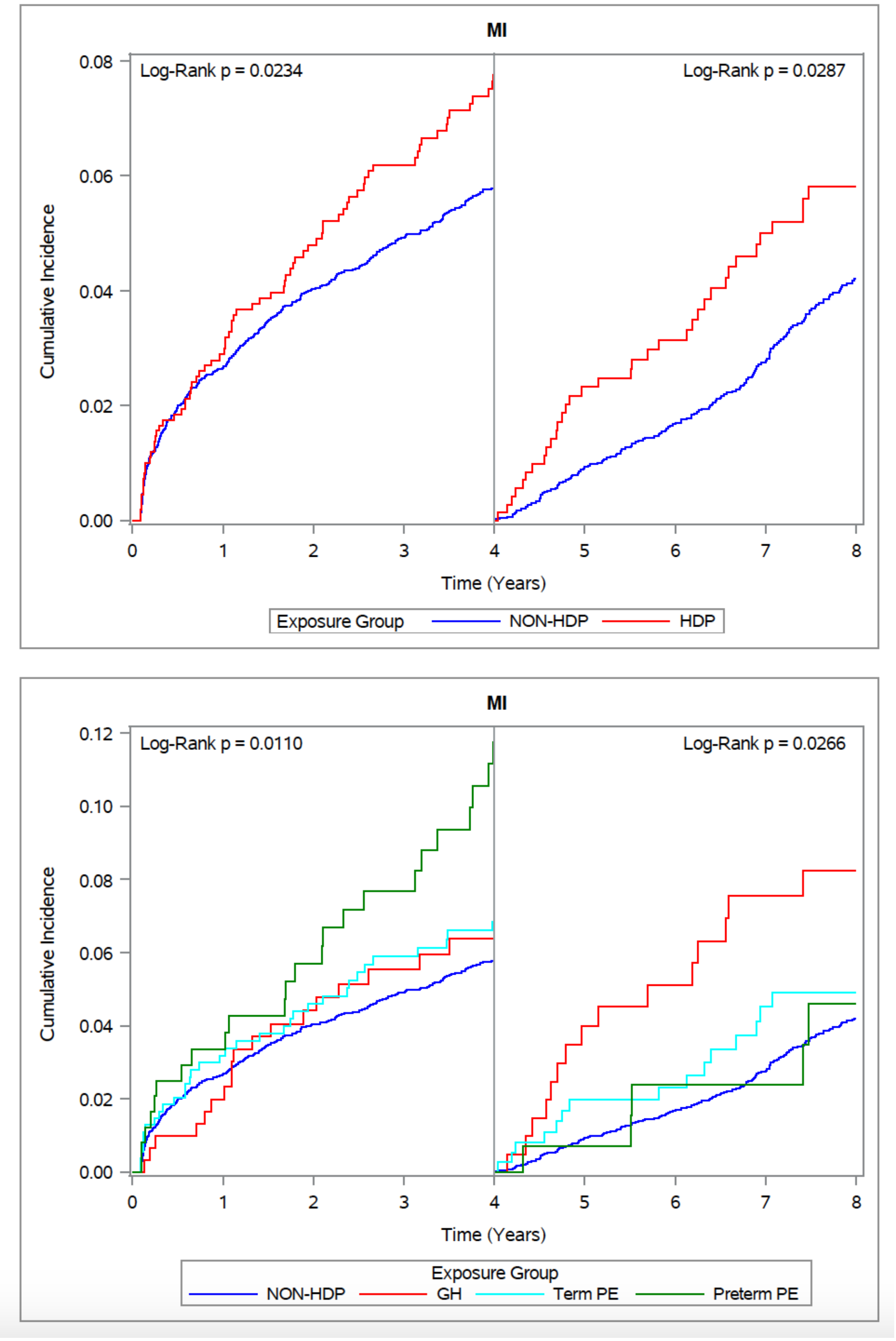

Figure S2. Cumulative incidence plot of myocardial infarctions following first coronary artery stenting in women by history of hypertensive disorders of pregnancy, with landmark analysis at four years and eight years.

Upper panel: Comparison according to history of hypertensive disorder of pregnancy.

Lower panel: Comparison according to subgroups of hypertensive disorder of pregnancy.

GH, gestational hypertension; HDP, hypertensive disorder of pregnancy; MI, myocardial infarction; non-HDP, no history of hypertensive disorders of pregnancy; PE, preeclampsia
